# Mechanistic Evaluation of Luteolin in Wound Healing of Diabetic Foot Ulcer via Nrf2 Signaling

**DOI:** 10.1002/ptr.70425

**Published:** 2026-08-07

**Authors:** Muhammed Yusuf, Ayfer Busra Benkli, Ramiz Yusuf

**Affiliations:** ^1^ Department of Pharmacology Anadolu University, Faculty of Pharmacy Eskisehir Turkey; ^2^ Department of Toxicology Yeditepe University, Faculty of Pharmacy Istanbul Turkey; ^3^ Faculty of Health Sciences, Department of Physiotherapy and Rehabilitation Kırıkkale University Kırıkkale Turkey

**Keywords:** diabetic foot ulcer network pharmacology, ferroptosis, luteolin, Nrf2 pathway, wound healing

## Abstract

Diabetic foot ulcers (DFUs) are a leading cause of lower‐limb amputation, with oxidative stress and ferroptosis playing critical roles in impaired wound healing. This study aimed to evaluate the therapeutic potential of luteolin (LU), a naturally occurring plant‐derived flavonoid, in DFU repair and to elucidate its underlying molecular mechanisms. A DFU rat model was established using a high‐fat/high‐glucose diet combined with streptozotocin injection, followed by full‐thickness skin wounding. LU was topically administered at different concentrations. Wound closure was monitored macroscopically, while histopathological changes, inflammatory cytokines, and growth factor levels were assessed by microscopy and ELISA. Network pharmacology and molecular docking analyses were performed to identify potential targets and signaling pathways, which were subsequently validated in vitro using human umbilical vein endothelial cells (HUVECs). LU significantly accelerated wound closure in a dose‐dependent manner, accompanied by reduced oxidative stress and pro‐inflammatory cytokine production, enhanced collagen deposition, increased angiogenesis, and upregulated expression of key growth factors. Bioinformatics analysis identified ferroptosis inhibition and activation of the nuclear factor erythroid 2–related factor 2 (Nrf2) signaling pathway as central mechanisms, which were confirmed experimentally. LU reduced intracellular reactive oxygen species levels and lipid peroxidation and promoted HUVEC migration and viability. Notably, pharmacological inhibition of Nrf2 with ML385 markedly reversed the protective effects of LU, indicating a pivotal role for Nrf2 signaling. Collectively, these findings demonstrate that LU promotes DFU healing through activation of Nrf2 signaling and suppression of ferroptosis, supporting its potential as a therapeutic candidate for the management of diabetic wounds.

## Graphical abstract

Proposed mechanism by which Luteolin (LU) promotes diabetic foot ulcer healing. LU attenuates hyperglycemia‐induced oxidative stress and lipid peroxidation, improves endothelial cell viability, and suppresses ferroptosis through Nrf2‐dependent antioxidant signaling, thereby accelerating angiogenesis and wound repair.

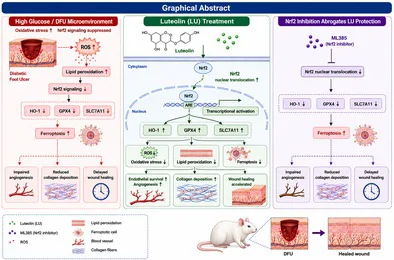

## 1 Introduction

Diabetic foot ulcer (DFU) is one of the most debilitating complications of diabetes mellitus, frequently resulting in poor quality of life, recurrent hospitalizations, and, in severe cases, limb amputation (Armstrong et al. 2017; Zhang et al. 2017). The impaired healing process in DFU is multifactorial, involving persistent hyperglycemia, neuropathy, infection, and microvascular dysfunction, all of which contribute to delayed wound closure (Noor et al. 2015; Guo and DiPietro 2010). A central mechanism underlying this pathology is the excessive production of reactive oxygen species (ROS) and the inability of endogenous antioxidant defenses to restore redox balance (Burgess et al. 2021). This uncontrolled oxidative stress damages lipids, proteins, and DNA, thereby aggravating chronic inflammation and impairing angiogenesis, ultimately resulting in defective tissue repair (Schäfer and Werner 2008).

Emerging evidence has highlighted the role of ferroptosis, a regulated, iron‐dependent form of cell death, in diabetic complications (Stockwell et al. 2017). Depletion of glutathione peroxidase 4 (GPX4), a crucial enzyme that detoxifies lipid hydroperoxides, and accumulation of lipid peroxides are hallmarks of ferroptosis (Yang et al. 2014). In DFU, increased iron deposition and reduced antioxidant capacity promote ferroptotic injury of vascular and epidermal cells, further exacerbating the delay in wound healing (Wang et al. 2024; Feng et al. 2022). Thus, suppression of ferroptosis has been proposed as a promising therapeutic approach to accelerate cutaneous repair in diabetic conditions.

Nuclear factor erythroid 2–related factor 2 (Nrf2) is a transcription factor that promotes the expression of detoxifying and cytoprotective genes, which in turn control the cellular defense response against oxidative stress (Ma 2013). Kelch‐like ECH‐associated protein 1 (Keap1) maintains Nrf2's cytoplasmic restriction while at rest. NAD(P)H: glutamate–cysteine ligase (GCL), quinone oxidoreductase 1 (NQO1), and heme oxygenase‐1 (HO‐1) are among the genes whose transcription is triggered by the antioxidant response element (ARE) when Nrf2 translocates to the nucleus in response to oxidative stimulation (Itoh et al. 1999; Kensler et al. 2007). Beyond classical antioxidant effects, Nrf2 activation plays an essential role in regulating ferroptosis by preserving GPX4 activity and glutathione (GSH) levels (Dodson et al. 2019). Previous work has shown that pharmacological activation of Nrf2 accelerates wound closure, enhances angiogenesis, and attenuates inflammatory signaling in diabetic models (Long et al. 2016; Xu et al. 2018). Luteolin (LU), a naturally occurring flavonoid found in many fruits and vegetables, has been widely studied for its anti‐inflammatory, antioxidant, and anti‐apoptotic properties (Seelinger et al. 2008). In various experimental models, LU has demonstrated protective effects against oxidative stress–mediated injury by scavenging ROS, modulating intracellular signaling pathways, and maintaining mitochondrial function (López‐Lázaro 2009; Aziz et al. 2018). Importantly, recent reports suggest that LU can regulate ferroptosis by modulating the expression of Nrf2 and its downstream targets (Xiang et al. 2025). Previous studies have demonstrated that LU exerts antidiabetic, antioxidant, and anti‐inflammatory effects in streptozotocin (STZ)‐induced diabetic models, contributing to improved glycemic control and attenuation of oxidative stress–mediated tissue injury. Kahksha et al. (2023) and related studies have provided evidence that LU mitigates hyperglycemia‐associated oxidative damage and modulates key molecular pathways relevant to diabetic complications. These findings establish a strong experimental foundation for luteolin's therapeutic potential in diabetes‐associated pathologies. However, despite these well‐documented antidiabetic and antioxidant properties, the specific role of LU in diabetic foot ulcer repair, particularly its mechanistic involvement in Nrf2‐regulated ferroptosis during wound healing, has not been systematically investigated. The present study was designed to evaluate the wound‐healing potential of LU in a streptozotocin (STZ)‐induced diabetic rat model and in high‐glucose–challenged human umbilical vein endothelial cells (HUVECs). We aimed to investigate whether LU promotes wound repair through Nrf2‐dependent suppression of ferroptosis. To test this hypothesis, we assessed wound closure dynamics, angiogenesis, inflammatory status, and oxidative stress in vivo, as well as ROS production, lipid peroxidation, and ferroptosis‐related protein expression in vitro. Furthermore, the pharmacological inhibitor ML385 was used to confirm the specific involvement of Nrf2 signaling.

## 2 Materials and Methods

### 2.1 Materials and Reagents

Luteolin (LU; purity ≥ 98%; catalogue no. L9283), streptozotocin (STZ; catalogue no. S0130), sodium citrate tribasic dihydrate (catalogue no. S4641), citric acid monohydrate (catalogue no. C1909), recombinant human epidermal growth factor (rhEGF; catalogue no. E9644), and 2′,7′‐dichlorodihydrofluorescein diacetate (DCFH‐DA; catalogue no. D6883) were obtained from Sigma‐Aldrich (Merck KGaA, Darmstadt, Germany; Sigma‐Aldrich, St. Louis, Missouri, USA). Citrate buffer was prepared using sodium citrate and citric acid, adjusted to the required pH, and used immediately to prepare the STZ solution. The selective Nrf2 inhibitor ML385 (catalog no. GC19254) was purchased from GLPBIO (Montclair, California, USA).

Human umbilical vein endothelial cells (HUVECs; catalog no. 8000) and Endothelial Cell Medium (ECM; catalog no. 1001) were obtained from ScienCell Research Laboratories (Carlsbad, California, USA). According to the supplier's quality‐control documentation, the cells were characterized by their cobblestone morphology, uptake of acetylated low‐density lipoprotein, and positive immunofluorescence staining for von Willebrand factor/Factor VIII and CD31/PECAM1. The supplied cells were certified negative for mycoplasma, bacteria, yeast, fungi, HIV‐1, hepatitis B virus, and hepatitis C virus.

Trypsin–EDTA solution (0.25%; catalog no. 0103) was obtained from ScienCell Research Laboratories. Cell viability was assessed using Cell Counting Kit‐8 (CCK‐8; product code CK04; Dojindo Laboratories, Kumamoto, Japan). Corning Matrigel Basement Membrane Matrix, LDEV‐free (catalog no. 354234), and sterile Corning Transwell inserts containing 6.5‐mm, 8.0‐μm‐pore polycarbonate membranes (catalog no. 3422) were obtained from Corning Incorporated (Corning, New York, USA).

Intracellular lipid peroxidation was assessed using the BODIPY 581/591 C11 Lipid Peroxidation Sensor (catalog no. D3861; Invitrogen, Thermo Fisher Scientific, Waltham, Massachusetts, USA). Intracellular reactive oxygen species were detected using DCFH‐DA (catalog no. D6883; Sigma‐Aldrich).

For immunoblotting, rabbit anti‐Nrf2 antibody (catalogue no. SAB4501984; 1:1000), rabbit anti‐HO‐1 antibody (catalogue no. H4535; 1 μg/mL), rabbit anti‐GPX4 antibody (catalogue no. SAB5700944; 1:1000), rabbit anti‐SLC7A11 antibody (catalogue no. SAB5700735; 1:1000), and mouse monoclonal anti‐β‐actin antibody, clone AC‐15 (catalogue no. A5441; 1:5000), were obtained from Sigma‐Aldrich. CD34 immunohistochemistry was performed using a recombinant rabbit monoclonal anti‐CD34 antibody, clone 5C15 (catalog no. ZRB1232; 1:1000; Sigma‐Aldrich). The secondary antibodies were HRP‐conjugated goat anti‐rabbit IgG (catalog no. A0545; 1:80,000 for immunoblotting and 1:200 for immunohistochemistry) and HRP‐conjugated rabbit anti‐mouse IgG (catalog no. A9044; 1:80,000), both obtained from Sigma‐Aldrich.

Rat TNF‐α (catalog no. RAB0480), IL‐6 (catalog no. RAB0312), VEGF‐A (catalog no. RAB0512), EGF (catalog no. RAB0567), and PDGF‐BB (catalog no. RAB1152) ELISA kits were obtained from Sigma‐Aldrich. The manufacturer‐reported analytical characteristics were as follows: TNF‐α, standard‐curve range 82.3–20,000 pg/mL and sensitivity 25 pg/mL; IL‐6, 40.96–10,000 pg/mL and sensitivity 15 pg/mL; VEGF‐A, 0.82–200 pg/mL and sensitivity 2 pg/mL; EGF, 0.82–200 pg/mL and sensitivity 0.8 pg/mL; and PDGF‐BB, 8.19–2000 pg/mL and sensitivity 8 pg/mL. For these kits, the manufacturer reported intra‐assay coefficients of variation below 10% and inter‐assay coefficients of variation below 12%. All ELISA measurements were performed according to the manufacturer's instructions.

For preparation of the topical formulations, LU was dissolved in DMSO at concentrations of 50, 100, and 200 μg/mL. One milliliter of each solution containing 50, 100, or 200 μg LU, respectively, was uniformly incorporated into 16 g of petroleum jelly to prepare the low‐, medium‐, and high‐concentration formulations. The corresponding nominal LU concentrations were approximately 3.125, 6.25, and 12.5 μg/g of petroleum jelly for the LU‐L, LU‐M, and LU‐H formulations, respectively. The formulations were prepared under identical conditions and stored at 4°C until use.

The calculations are:

$$\begin{array}{c} 50\;\mathrm{\mu g}÷16\;g=\frac{3.125\;\mathrm{\mu g}}{g}\\ 100\;\mathrm{\mu g}÷16\;g=\frac{6.25\;\mathrm{\mu g}}{g}\\ 200\;\mathrm{\mu g}÷16\;g=12.5\;\mathrm{\mu g}/g \end{array}$$

These values assume that the formulation concentration was calculated relative to the 16 g petroleum‐jelly base.

### 2.2 Animals

Male Sprague–Dawley rats aged 8–10 weeks were obtained from the Anadolu University Experimental Animal Center (Eskişehir, Türkiye). Animals were housed under controlled environmental conditions (22°C ± 2°C; 12‐h light/dark cycle) with unrestricted access to standard laboratory rodent chow and drinking water. All experimental procedures were reviewed and approved by the Anadolu University Animal Experiments Local Ethics Committee (approval no. 2021‐36) and were conducted in accordance with the approved institutional protocol. The study is reported in accordance with the ARRIVE 2.0 guidelines (Sert et al. 2020; Lilley et al. 2020).

### 2.3 DFU Model Establishment, Sample Size, and Group Allocation

Following a 1‐week acclimatization period, 60 male Sprague–Dawley rats were allocated equally to six experimental groups, with 10 animals in each group: control, DFU model, recombinant human epidermal growth factor (rhEGF), low‐dose luteolin perchlorate (LU‐L), medium‐dose luteolin perchlorate (LU‐M), and high‐dose luteolin perchlorate (LU‐H). No formal priori power calculation was performed. The group size was selected based on previously published studies using comparable streptozotocin‐induced diabetic wound models and was considered adequate to detect biologically meaningful treatment effects while limiting animal use in accordance with the reduction principle (Tang et al. 2025; Xu et al. 2024).

Animals were allocated to experimental groups before treatment initiation. Because the original experimental records did not specify the use of a computer‐generated randomization sequence, the exact method of random allocation could not be confirmed. Similarly, formal blinding during treatment administration was not documented. Wound‐area measurements, histological assessment, immunohistochemical quantification, and image analysis were performed using coded samples wherever possible; however, the absence of fully documented allocation concealment and blinding is acknowledged as a methodological limitation.

Except for animals in the control group, all rats received a high‐fat, high‐sugar diet for 30 days. Diabetes was induced by a single intraperitoneal injection of streptozotocin at 40 mg/kg, freshly prepared in sodium citrate buffer. Control animals received an equivalent volume of sodium citrate buffer. Fasting blood glucose concentrations were measured for three consecutive days using a portable glucometer. Diabetes was considered successfully established when fasting blood glucose was ≥ 16.7 mmol/L and was accompanied by clinical signs such as weight loss, polyuria, and polydipsia.

After confirmation of diabetes, the dorsal hair was removed, and animals were anesthetized with isoflurane. A full‐thickness circular excisional wound measuring 15 mm in diameter was created on the dorsal skin under aseptic conditions. Animals subsequently received their assigned treatments according to the experimental protocol. The control and DFU model groups received the corresponding vehicle formulation, the positive‐control group received topical rhEGF, and the LU treatment groups received the low‐, medium‐, or high‐concentration LU formulation.

Following wound induction, 0.2 g of the assigned formulation was applied evenly to the entire wound surface once daily until the designated terminal assessment on day 7 or day 14. Based on the nominal formulation concentrations, each application delivered approximately 0.625 μg per wound in the LU‐L group, 1.25 μg per wound in the LU‐M group, and 2.50 μg per wound in the LU‐H group. The control and model groups received an equivalent amount of vehicle formulation, whereas the positive‐control group received rhEGF according to the specified treatment regimen. Because LU was administered topically to a standardized 15‐mm wound, the dose was expressed as micrograms per wound per application rather than milligrams per kilogram of body weight.

Wound closure was assessed on days 0, 3, 7, 10, and 14. Animals designated for terminal tissue collection were deeply anesthetized before sample collection and euthanasia on days 7 or 14. At the designated terminal time points on days 7 and 14, animals were deeply anesthetized with isoflurane. Blood was collected from the abdominal aorta, after which euthanasia was completed by isoflurane overdose. Wound‐edge skin, granulation tissue, and other required samples were then collected for subsequent analyses. The exact number of animals used for each terminal time point and individual analysis was reported in the corresponding figure legends. Animal mortality, exclusions, and the final number of animals included in each analysis were documented. No animal or sample was excluded from the analysis unless a predefined technical or humane‐endpoint criterion was met.

### 2.4 Wound Healing Assessment

Wound healing was recorded on days 0, 3, 7, 10, and 14.

$$\text{Wound closure}\;\left(\%\right)=\frac{A\;0-\mathrm{At}}{A\;0}\times 100$$

where $A_{0}$represents the wound area on day 0 and $A_{t}$represents the wound area at the indicated assessment time.

ImageJ software (NIH) was used for area measurement.

### 2.5 Histological Evaluation

Every group was randomly assigned at least three rats for histological examination on days 7 and 14. Following paraffin embedding, skin samples were sectioned, fixed, and stained with Masson's trichrome and hematoxylin–eosin (HE). A microscopic examination of tissue morphology and collagen deposition was conducted.

### 2.6 Immunohistochemistry

Sections underwent dewaxing, hydration, antigen retrieval, blocking, and incubation with primary antibody (CD34, 1:200) and secondary antibody (1:500). DAB chromogen and hematoxylin counterstain were used for detection. The three most vascularized regions at 50× were identified, and five random fields at 200 × magnification were assessed for microvessel density.

### 2.7 Terminal Blood Collection, Serum Preparation, and ELISA Assays

On days 7 and 14, animals designated for sample collection were deeply anesthetized with isoflurane. Blood was collected from the abdominal aorta as a terminal procedure immediately before euthanasia. This route was selected because it provided sufficient blood volume for the simultaneous measurement of multiple inflammatory cytokines, growth factors, and oxidative stress‐related biomarkers.

Blood was collected into anticoagulant‐free tubes and allowed to clot for 30 min at room temperature. The samples were then centrifuged at 3000 rpm for 15 min at 4°C. The resulting serum fraction was carefully transferred into labeled microcentrifuge tubes, divided into aliquots, and stored at −80°C until analysis. Repeated freeze–thaw cycles were avoided.

Serum concentrations of tumor necrosis factor‐α (TNF‐α), interleukin‐6 (IL‐6), vascular endothelial growth factor (VEGF), epidermal growth factor (EGF), platelet‐derived growth factor (PDGF), and reactive oxygen species‐related biomarkers were measured using commercial ELISA kits according to the manufacturers' instructions. Absorbance was measured at the wavelength specified for each kit, and analyte concentrations were calculated from the corresponding standard curves.

### 2.8 Network Pharmacology and R‐Based Bioinformatics Analysis

The canonical SMILES structure of luteolin perchlorate (LU) was retrieved from PubChem and submitted independently to Super‐PRED, SwissTargetPrediction, and PharmMapper for prediction of potential protein targets. All searches were restricted to *Homo sapiens* . The target lists generated by the three databases were exported and processed using R version 4.6.0.

Protein identifiers were standardized to official human gene symbols using the AnnotationDbi and org.Hs.eg.db packages. Unmapped entries and duplicate records within each database were removed. Because the three target‐prediction platforms use different scoring systems that are not directly comparable, a single universal numerical score threshold was not applied. Instead, a cross‐database consensus filter was used: only targets independently identified by at least two of the three prediction databases were retained for subsequent analyses. This approach was used to reduce database‐specific false‐positive predictions and improve target reliability.

Genes associated with diabetic foot ulcers were collected from GeneCards, OMIM, the Therapeutic Target Database, and DisGeNET. Ferroptosis‐associated genes were obtained from FerrDb. All disease‐ and ferroptosis‐related gene lists were standardized to official human gene symbols, and duplicate entries were removed in R. Common targets shared among LU, diabetic foot ulcer, and ferroptosis were identified using set‐intersection functions in R and visualized using the ggVennDiagram package.

The intersecting targets were submitted to the STRING database for protein–protein interaction analysis, with the species restricted to *Homo sapiens* . Interactions meeting a minimum combined confidence score of 0.400 were retained. The resulting interaction data were imported into R and analyzed using the igraph package. Network‐topology parameters, including degree, betweenness, and closeness centrality, were calculated. Core targets were prioritized primarily according to degree centrality, with betweenness and closeness centrality used as supporting measures.

Gene Ontology enrichment analysis, including biological process, cellular component, and molecular function categories, was performed using the clusterProfiler package. KEGG and WikiPathways enrichment analyses were conducted using enrichKEGG and enrichWP, respectively. Gene identifiers were converted to Entrez IDs before enrichment analysis. *p* values were adjusted using the Benjamini–Hochberg method, and terms with adjusted *p* < 0.05 were considered statistically significant. Enrichment results were visualized using the enrichplot and ggplot2 packages.

### 2.9 Molecular Docking and R‐Based Processing of Docking Results

The three‐dimensional structure of LU was obtained from PubChem and subjected to energy minimization using ChemOffice. The optimized ligand structure was converted to PDB format and subsequently prepared in AutoDockTools version 1.5.6. Polar hydrogen atoms were added, nonpolar hydrogen atoms were merged, Gasteiger charges were assigned, and the ligand was saved in PDBQT format.

Core target proteins were selected based on network‐topology analysis, enrichment results, biological relevance to diabetic wound healing and ferroptosis, and the availability of suitable experimentally determined protein structures. The three‐dimensional structures of PTGS2, HIF1A, PPARG, and NFE2L2 were obtained from the RCSB Protein Data Bank.

Protein structures were prepared using PyMOL by removing crystallographic water molecules, unrelated ligands, and nonessential heteroatoms. Polar hydrogen atoms and Gasteiger charges were subsequently added using AutoDockTools, and the prepared receptor structures were converted to PDBQT format.

Molecular docking was performed using AutoDock Vina. The docking search space for each target was defined around the known or predicted ligand‐binding pocket. For each target, multiple ligand poses were generated, and the pose with the lowest predicted Vina binding‐energy score located within the predefined binding pocket was selected for further evaluation.

Predicted protein–ligand interactions, including hydrogen bonds and hydrophobic contacts, were examined and visualized using PyMOL. Docking‐score outputs were imported into R version 4.6.0 and processed using the readr, dplyr, and ggplot2 packages. R was used only to organize, rank, summarize, and visualize the docking results; the docking calculations themselves were performed using AutoDock Vina.

The docking results were interpreted as computational and hypothesis‐generating predictions rather than experimental confirmation of direct protein binding.

### 2.10 Screening for Viability and Cell Culture

Complete ECM medium was used to cultivate HUVECs at 37°C with 5% CO_2_. The cells were passaged when they reached 80% confluence. Ideal LU concentrations were identified using the CCK‐8 assay and classified as low, medium, and high. Experimental cells were treated with 30 mM glucose, while controls received 5 mM.

### 2.11 Cell Viability and Grouping

Cells were divided into blank control, high‐glucose model, LU (optimal dose), and LU + ML385 co‐treatment groups. CCK‐8 assays were used to evaluate cell viability under each condition.

### 2.12 Migration and Angiogenesis Assays

Scratch Assay: HUVECs grew to 90% of the confluence in 6‐well plates. A 100 μL pipette tip was used to create scratches, and cells were treated with glucose ± LU. Images were captured at 0 and 24 h.

### 2.13 Transwell Assay

Cells were seeded in Transwell chambers with or without LU and glucose. After incubation, cells were fixed, stained, and counted under a microscope.

### 2.14 Assay for Tube Formation

After being seeded onto wells coated with Matrigel, the cells were incubated for 6 h. Structures resembling capillaries were seen and measured.

### 2.15 ROS and Lipid Peroxidation Assays

ROS Detection: DCFH‐DA (10 μM) was used to detect intracellular ROS. Following incubation, cells were washed and imaged under a fluorescence microscope.

### 2.16 Lipids Undergo Peroxidation

BODIPY 568/594 C11 dye was applied at 5 μM and incubated with cells for 30 min. Oxidized (green) and reduced (red) fluorescence signals were visualized using confocal microscopy and analyzed with ImageJ.

### 2.17 Protein Expression Analysis

Protein expression levels of HO‐1, GPX4, SLC7A11, and Nrf2 were quantified following the respective experimental treatments. Relative protein expression values were normalized to the control group and analyzed using Image Pro Plus 6.0 (Media Cybernetics, Rockville, MD, USA). The resulting quantitative data were used for statistical analysis.

### 2.18 Statistical Analysis

Statistical analyses were performed using R version 4.6.0. Data were presented as mean ± (SD/SEM), and the number of independent biological replicates or animals included in each analysis is reported in the corresponding figure legend. Data distribution and homogeneity of variance were evaluated using the Shapiro–Wilk and Levene tests, respectively.

Over time, wound‐closure data collected repeatedly from the same animals were analyzed using two‐way repeated‐measures analysis of variance (ANOVA), with treatment group and time as factors, followed by an appropriate multiplicity‐adjusted post hoc test. Comparisons involving three or more independent groups at a single time point were analyzed using one‐way ANOVA followed by Dunnett's multiple‐comparison test against the relevant model or high‐glucose group. Two group comparisons, where applicable, were analyzed using a two‐tailed unpaired Student's *T*‐test. A two‐sided *p* value < 0.05 was considered statistically significant.

## 3 Results

### 3.1 Analysis of the Rate and Progress of Wound Healing in DFU Rats

Macroscopic changes from day 0 to day 14 are shown in Figure 1A,B, which show the wound healing process across various experimental groups. There were no appreciable variations in wound sizes between the groups at the beginning (day 0). In contrast to the treatment and control groups, the model group displayed a noticeably delayed healing trajectory, even though all groups showed decreases in wound area over the days. By day 14, while the model group still displayed marked inflammation and incomplete healing, the remaining groups, particularly those receiving LU, had entered the tissue remodeling phase with visible re‐epithelialization. Among these, the high‐dose LU group (LU‐H) exhibited the most pronounced improvement.

**FIGURE 1:**
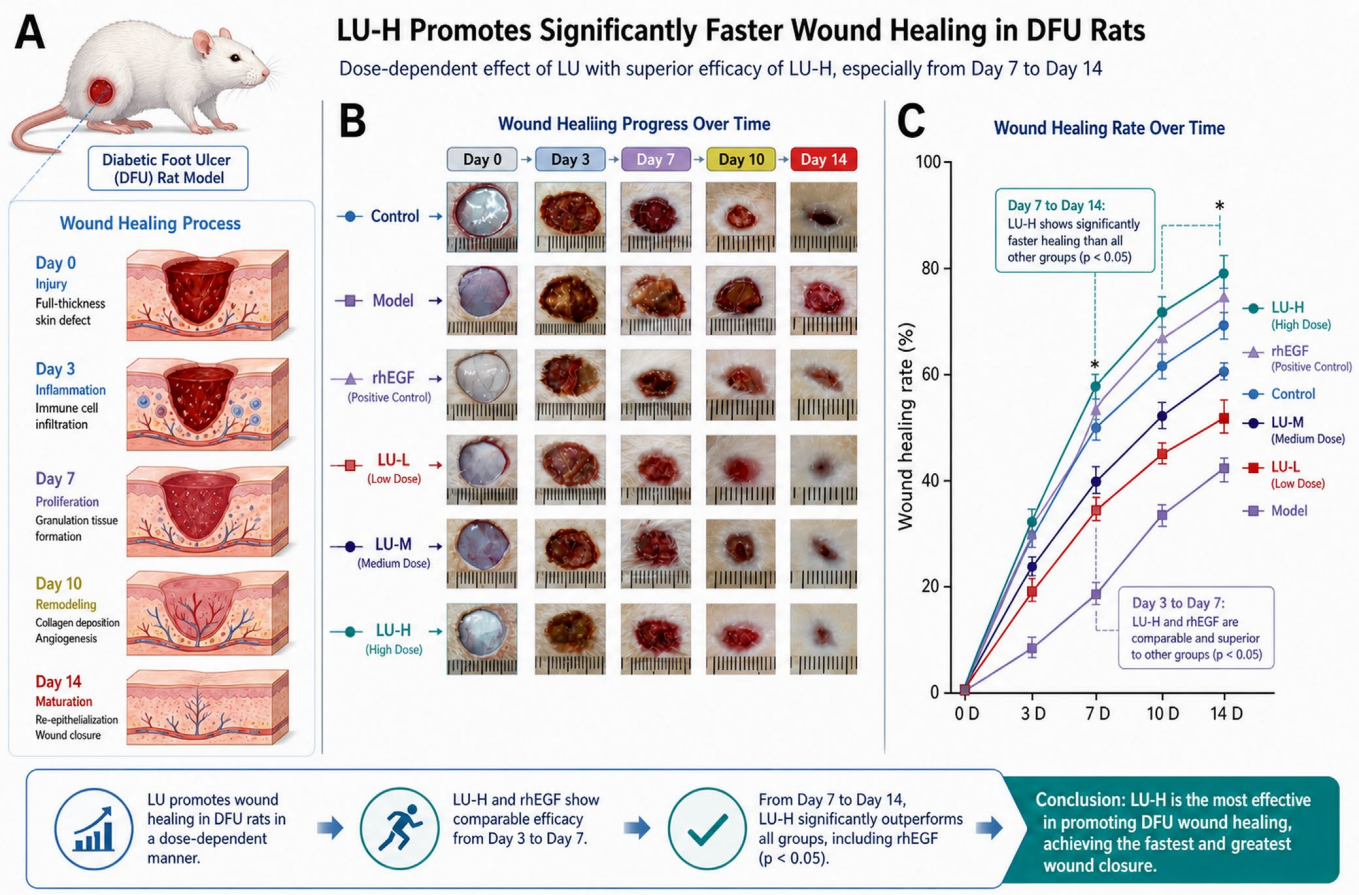
(A) Schematic representation of the diabetic foot ulcer rat model and the principal stages of wound repair from day 0 to day 14, including injury, inflammation, proliferation, remodeling, and maturation. (B) Representative photographs showing wound‐healing progression in the control, diabetic foot ulcer model, recombinant human epidermal growth factor (rhEGF), low‐dose luteolin (LU‐L), medium‐dose luteolin (LU‐M), and high‐dose luteolin (LU‐H) groups on days 0, 3, 7, 10, and 14. (C) Quantitative analysis of wound closure at the indicated time points. Wound closure was calculated relative to the initial wound area on day 0. LU accelerated wound healing in a dose‐dependent manner. The LU‐H and rhEGF groups showed comparable wound closure between days 3 and 7 and greater healing than the model, LU‐L, and LU‐M groups. From day 7 to day 14, LU‐H produced significantly greater wound closure than all other groups, including the rhEGF group. Data are presented as mean ± SD (*n* = 3). Statistical analysis was performed using two‐way repeated‐measures ANOVA followed by multiplicity‐adjusted pairwise comparisons. *p < 0.05 versus the indicated comparator.

### 3.2 LU Improves Histological Architecture and Collagen Deposition in DFU Wounds

Histological examination on day seven using hematoxylin and eosin (HE) staining (Figure 2) showed significant variations between the experimental groups. The control group showed some inflammatory cell infiltration, mild epidermal damage, and early neovascularization. The model group, on the other hand, showed signs of severe tissue disruption, such as cellular necrosis, eschar formation, large‐scale inflammatory infiltration, bleeding, and dermal collagen loss. Treatment with rhEGF and LU‐H preserved the epidermal structure and enhanced neovascularization, with the LU‐H group exhibiting a thicker stratum spinosum, an absence of abundant dermal collagen, and no inflammation. By day 14 (Figure 2), the control, rhEGF, and LU‐treated groups showed nearly complete epidermal regeneration and organized dermal collagen, whereas the model group retained structural deficits and inflammatory infiltration.

**FIGURE 2:**
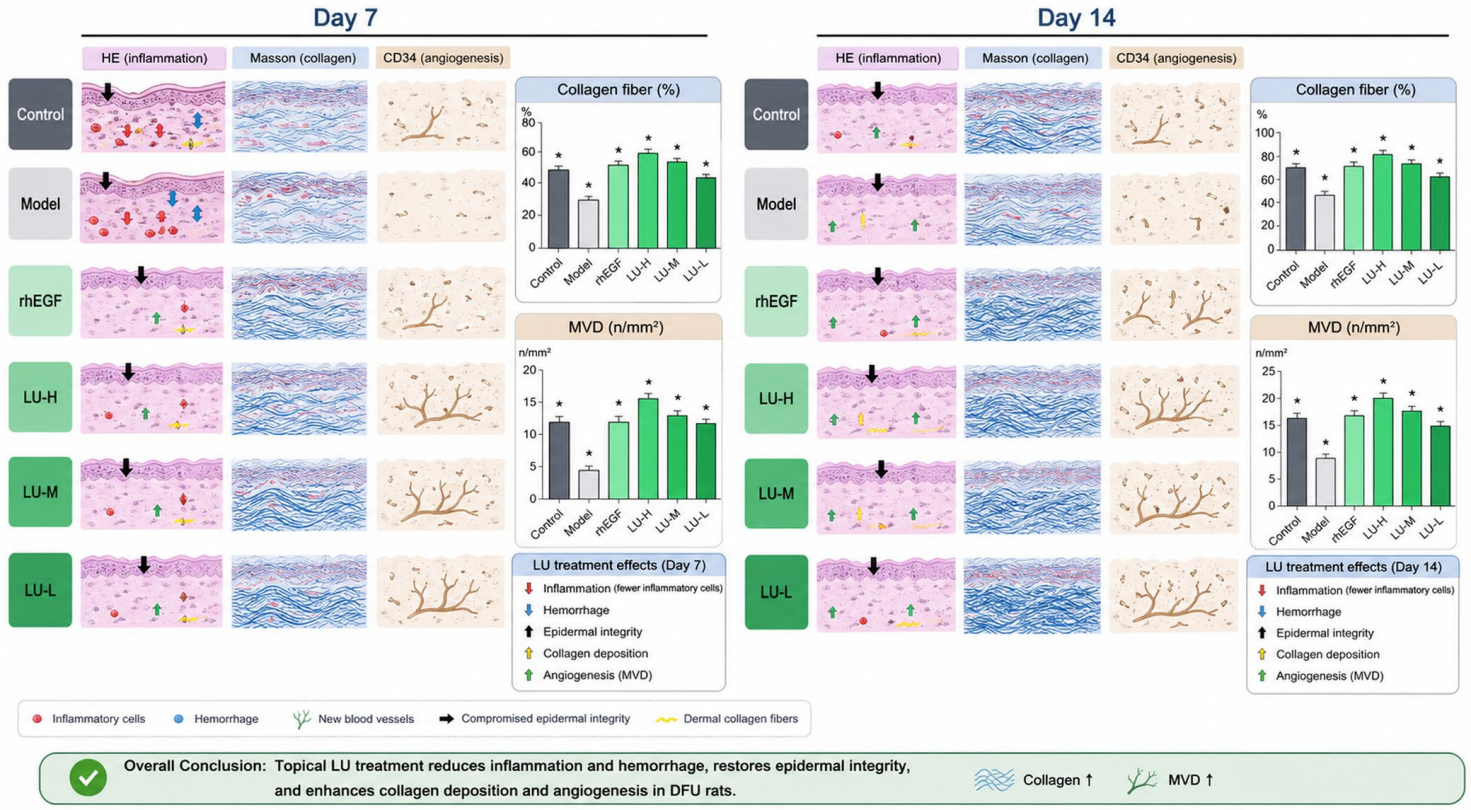
Topical LU improves histological architecture, collagen deposition, and angiogenesis in diabetic foot ulcer wounds. Representative histological and immunohistochemical images of wound tissues collected on days 7 and 14 from the control, DFU model, rhEGF, LU‐H, LU‐M, and LU‐L groups are shown. Hematoxylin and eosin (HE) staining illustrates tissue morphology and inflammatory changes, Masson's trichrome staining shows collagen deposition, and CD34 immunohistochemistry demonstrates angiogenesis and microvessel formation. In the HE panels, red arrows indicate inflammatory‐cell infiltration, blue arrows indicate hemorrhagic areas, green arrows indicate newly formed blood vessels, black arrows indicate compromised or restored epidermal integrity, and yellow arrows indicate dermal collagen fibers. Quantitative analysis of collagen fiber content (%) and microvessel density (MVD, n/mm^2^) is shown for days 7 and 14. Topical LU treatment reduced inflammation and hemorrhage, improved epidermal integrity, and enhanced collagen deposition and angiogenesis in a dose‐dependent manner, with the most pronounced effects observed in the LU‐H group. Data are presented as mean ± SD (*n* = 3 animals per group at each time point). Statistical comparisons at each time point were performed using one‐way ANOVA followed by Dunnett's multiple‐comparison test against the DFU model group. *p < 0.05, **p < 0.01, and ***p < 0.001 versus the DFU model group. Scale bar = 50 μm.

Masson's trichrome staining (Figure 2) showed abundant and well‐aligned collagen fibers in the dermis of the LU‐treated and rhEGF groups, in contrast to the disorganized and sparse collagen observed in the model group. Quantitative analysis (Figure 2) confirmed that the LU‐H group exhibited the highest collagen content at both points (*p* < 0.001 vs. model), with LU‐M, LU‐L, and rhEGF groups also showing significant improvements (*p* < 0.001). These results suggest that LU facilitates extracellular matrix remodeling by enhancing collagen deposition.

### 3.3 By Increasing Microvessel Density and CD34 Expression, LU Stimulates Angiogenesis

Using CD34 immunostaining, microvessel density (MVD) was measured to assess angiogenesis. Day 7 as well as Day 14 (Figure 2). MVD was considerably lower in the model group than in the control. All LU treatment groups, on the other hand, showed increased MVD; the LU‐H group, however, showed the strongest vascularization. Quantitative analysis (Figure 2) revealed that LU‐H significantly outperformed the rhEGF group in increasing MVD (*p* < 0.05), indicating enhanced neovascularization as part of LU's mechanism of wound repair.

### 3.4 LU Modulates Inflammatory Cytokines, Growth Factors, and Oxidative Stress Markers

ELISA results (Figure 3) demonstrated that IL‐6 and TNF‐α levels were markedly elevated in the model group at both day 7 and day 14, consistent with chronic inflammation in DFU wounds (Figure 3A,B). LU treatment significantly reduced these proinflammatory cytokines in a dose‐dependent manner. Conversely, the levels of VEGF, EGF, and PDGF (Figure 3C–E), which are critical for cell proliferation, migration, and angiogenesis, were suppressed in the model group but increased significantly in LU‐treated rats, especially in the LU‐H group.

**FIGURE 3:**
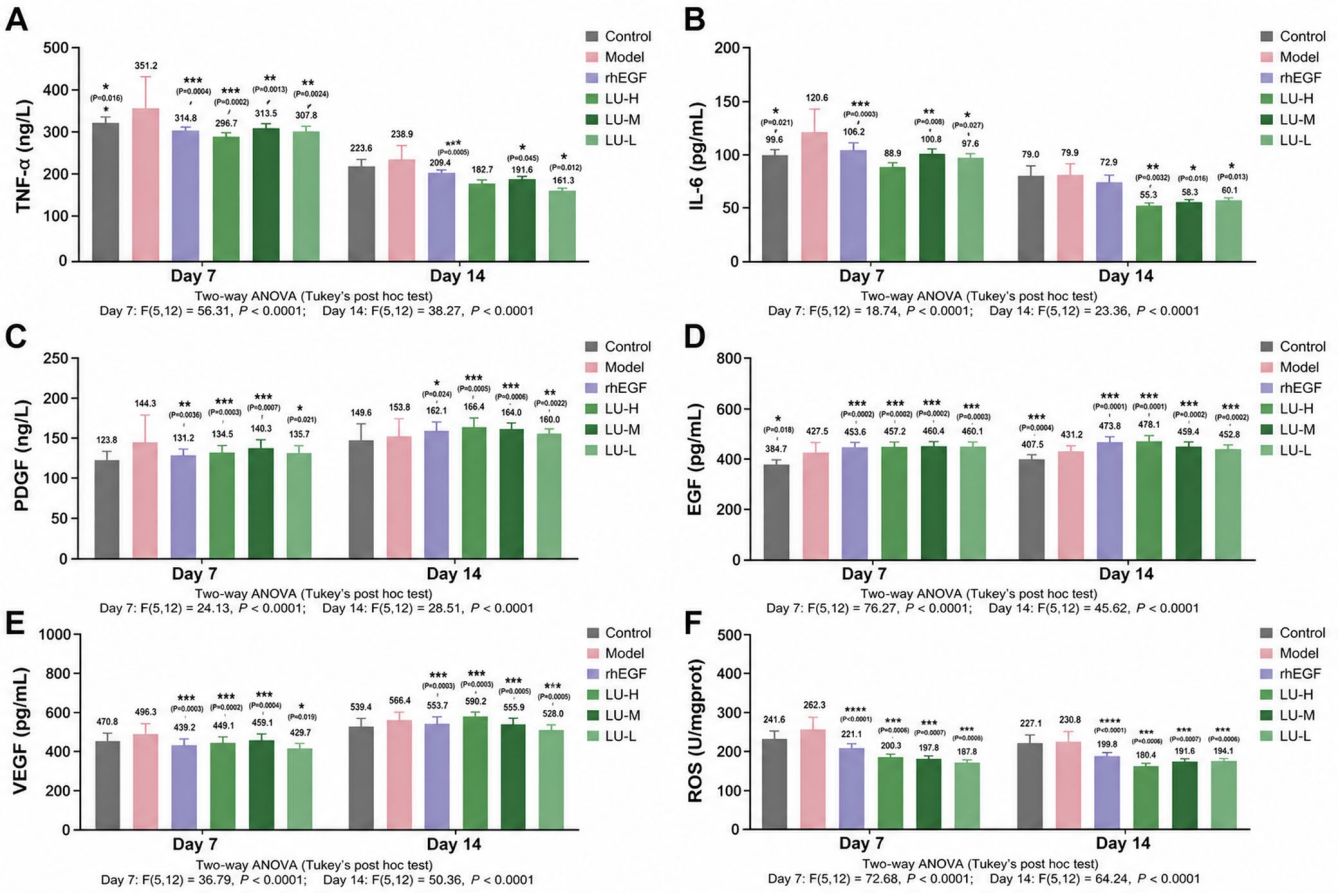
LU modulates inflammatory cytokines, growth factors, and oxidative stress in rats with diabetic foot ulcers. Levels of (A) tumor necrosis factor‐α (TNF‐α), (B) interleukin‐6 (IL‐6), (C) platelet‐derived growth factor (PDGF), (D) epidermal growth factor (EGF), (E) vascular endothelial growth factor (VEGF), and (F) ROS‐related activity were measured in samples collected on days 7 and 14 from the control, DFU model, recombinant human epidermal growth factor (rhEGF), high‐dose luteolin (LU‐H), medium‐dose luteolin (LU‐M), and low‐dose luteolin (LU‐L) groups. LU treatment reduced the elevated TNF‐α, IL‐6, and ROS‐related levels and increased PDGF, EGF, and VEGF levels relative to the untreated DFU model group. Data are presented as mean ± SD (*n* = 3 animals per group at each time point). Comparisons at each time point were performed using one‐way ANOVA followed by Dunnett's multiple‐comparison test against the DFU model group. Exact *p* values are shown above the corresponding bars; *p* < 0.05, *p* < 0.01, *p* < 0.001, and *p* < 0.0001 versus the DFU model group.

Additionally, ROS, an indicator of oxidative stress and ferroptosis, were elevated in model wounds at both time points (Figure 3F). LU administration significantly attenuated ROS levels in a dose‐responsive manner, suggesting that LU alleviates oxidative injury, possibly by modulating redox balance. These findings collectively support LU's role in regulating inflammatory responses, stimulating tissue regeneration, and mitigating oxidative damage in DFU.

### 3.5 Network Pharmacology Identifies Nrf2 as a Central Target in LU's Therapeutic Action

Computational analysis identified 778 potential LU targets using Super‐PRED and related databases. Cross‐referencing with 485 ferroptosis‐related genes and 3008 DFU‐associated genes revealed 28 common targets (Figure 4A). Protein–protein interaction (PPI) mapping (Figure 4B,C) highlighted PPARG, PTGS2, NFE2L2 (Nrf2), and HIF1A as key nodes with high degree values.

**FIGURE 4:**
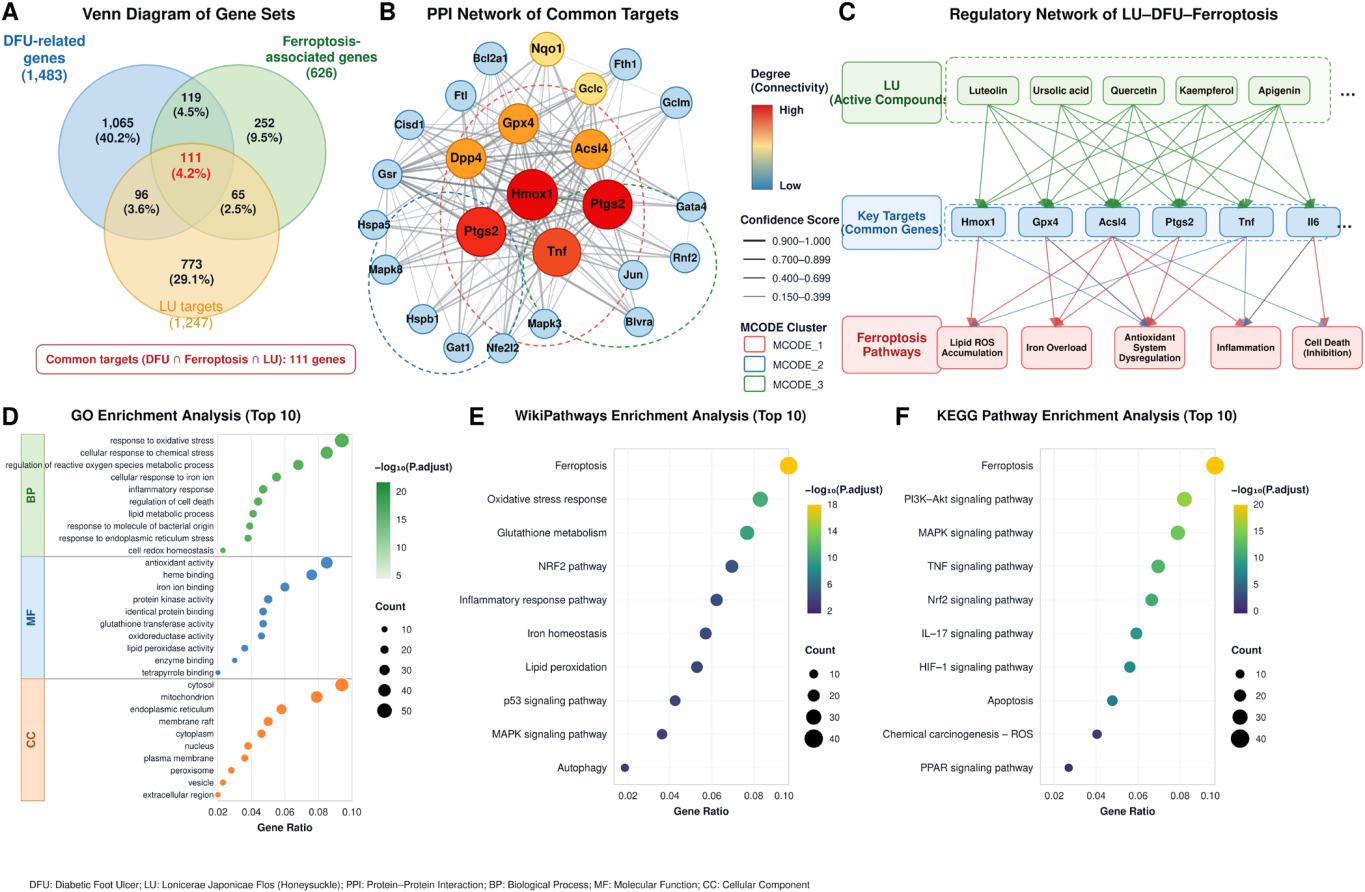
Network pharmacology analysis of the interaction among LU, diabetic foot ulcer, and ferroptosis. (A) Venn diagram showing the overlap among LU targets, diabetic foot ulcer (DFU)‐related genes, and ferroptosis‐associated genes. (B) Protein–protein interaction (PPI) network of the intersecting targets. Node color indicates degree (connectivity), and edge thickness reflects the confidence score of the interaction. (C) Regulatory network illustrating the relationships among LU, key common targets, and ferroptosis‐related biological processes. (D) Gene Ontology (GO) enrichment analysis of the common targets, including biological process (BP), molecular function (MF), and cellular component (CC) categories. (E) WikiPathways enrichment analysis of the common targets. (F) Kyoto Encyclopedia of Genes and Genomes (KEGG) pathway enrichment analysis of the common targets. These results suggest that LU may exert therapeutic effects in diabetic foot ulcer, at least in part, through modulation of ferroptosis‐related targets and pathways.

Gene Ontology (GO) analysis showed enrichment in biological processes related to oxidative stress response, hypoxic signaling, and inflammation. Molecular function annotations included ATP binding, protein binding, and kinase activity (Figure 4D). WikiPathway enrichment pointed strongly to Nrf2 signaling involvement (Figure 4E), while KEGG analysis suggested LU may act via pathways including AGE‐RAGE signaling, lipid metabolism, and ROS regulation (Figure 4F). These findings suggest that activation of the Nrf2 pathway and regulation of ferroptosis may be central to LU's mechanism in promoting DFU healing.

Although an initial broad target pool was generated, subsequent intersection analysis and PPI‐based prioritization substantially reduced complexity and enabled identification of core regulatory nodes relevant to DFU and ferroptosis.

### 3.6 Molecular Docking Suggests Potential Interactions Between LU and Selected Target Proteins

To provide structural support for the network‐pharmacology findings, molecular docking was performed to evaluate the predicted interactions of LU with PTGS2, PPARG, and KEAP1. Representative docking poses suggested that LU could occupy defined ligand‐binding pockets within these proteins and form putative hydrogen‐bond and polar interactions with selected amino acid residues (Figure 5). These findings represent hypothesis‐generating computational predictions and do not establish direct binding, target activation or inhibition, or the dynamic stability of the predicted complexes. The docking results were therefore interpreted cautiously and considered alongside the experimental evidence supporting Nrf2‐associated signaling.

**FIGURE 5:**
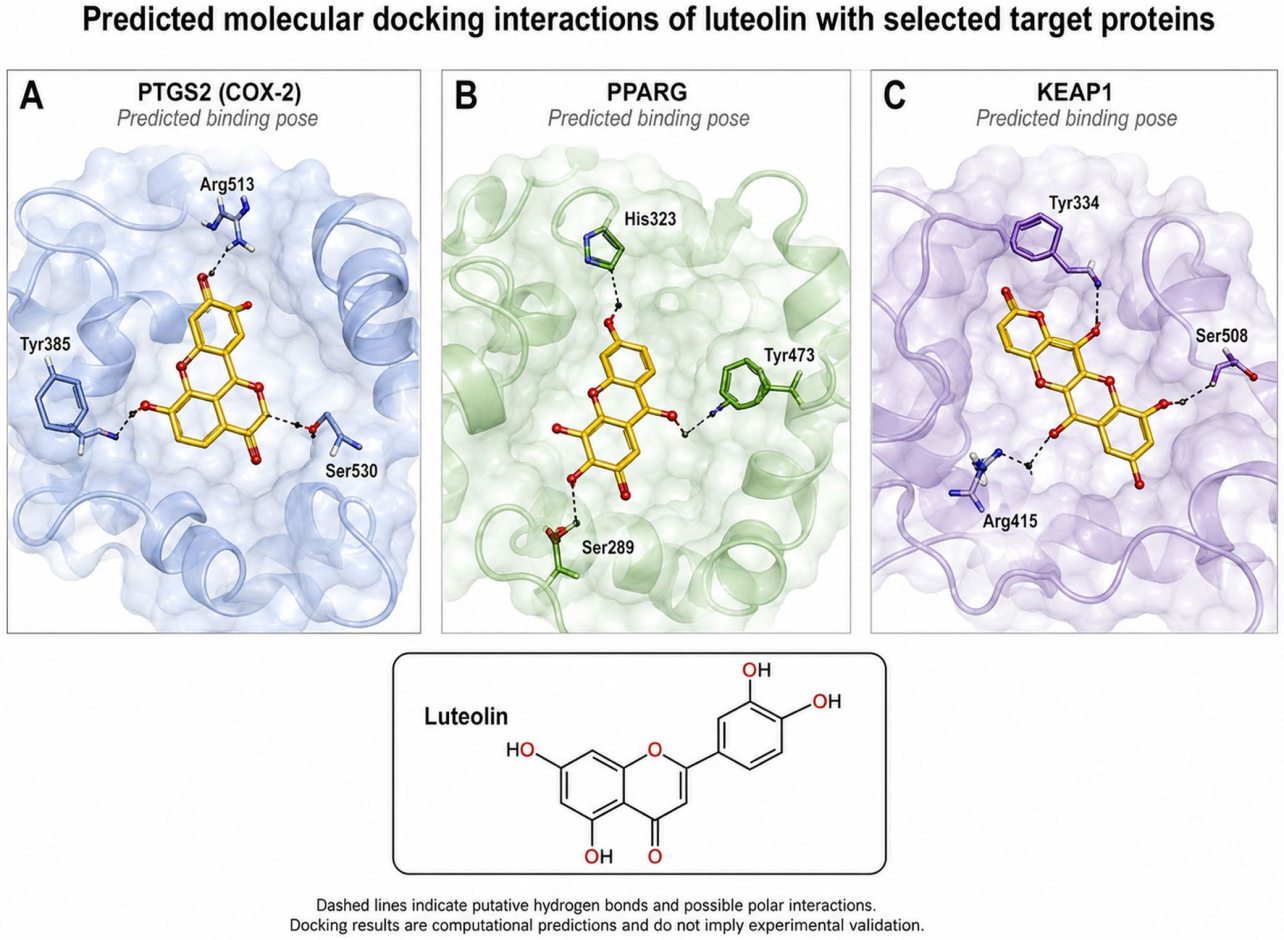
Predicted molecular docking interactions of LU with selected target proteins. Representative predicted binding poses of LU within the ligand‐binding pockets of (A) PTGS2/COX‐2, (B) PPARG, and (C) KEAP1 are shown. LU is presented in stick representation, whereas the target proteins are displayed as ribbon and semi‐transparent surface representations. Dashed lines indicate putative hydrogen‐bond or polar interactions between LU and selected amino acid residues. The docking poses were generated using AutoDock Vina and were interpreted as hypothesis‐generating predictions. These computational results do not constitute experimental confirmation of direct protein binding. Abbreviations: KEAP1, Kelch‐like ECH‐associated protein 1; PPARG, peroxisome proliferator‐activated receptor gamma; PTGS2, prostaglandin‐endoperoxide synthase 2; COX‐2, cyclooxygenase‐2.

Three LU concentrations were chosen for analysis using the CCK‐8 assay: 0.05 μM, 0.1 μM, and 0.2 μM (Figure 5A). Cell viability was significantly reduced in HUVECs exposed to high glucose (30 mM) compared with the normal glucose control (*p* < 0.001). Taken together with the enrichment analysis from the network pharmacology data, these results support the hypothesis that LU may promote DFU wound healing by regulating ferroptosis via the Nrf2 signaling pathway. To further validate these interactions functionally, subsequent in vitro experiments were conducted.

### 3.7 LU Enhances Cell Viability in a High‐Glucose Injury Model

Three LU concentrations were chosen for analysis using the CCK‐8 assay: 0.05 μM, 0.1 μM, and 0.2 μM (Figure 6A). Cell viability was significantly reduced in HUVECs exposed to high glucose (30 mM) in comparison to the normal glucose control (*p* < 0.001). However, treatment with all concentrations of LU significantly improved viability in the high‐glucose environment (*p* < 0.001 vs. HG group), with the LU‐treated groups showing a marked protective effect (Figure 6B). These findings suggest that LU attenuates high‐glucose‐induced cytotoxicity and improves endothelial cell survival.

**FIGURE 6:**
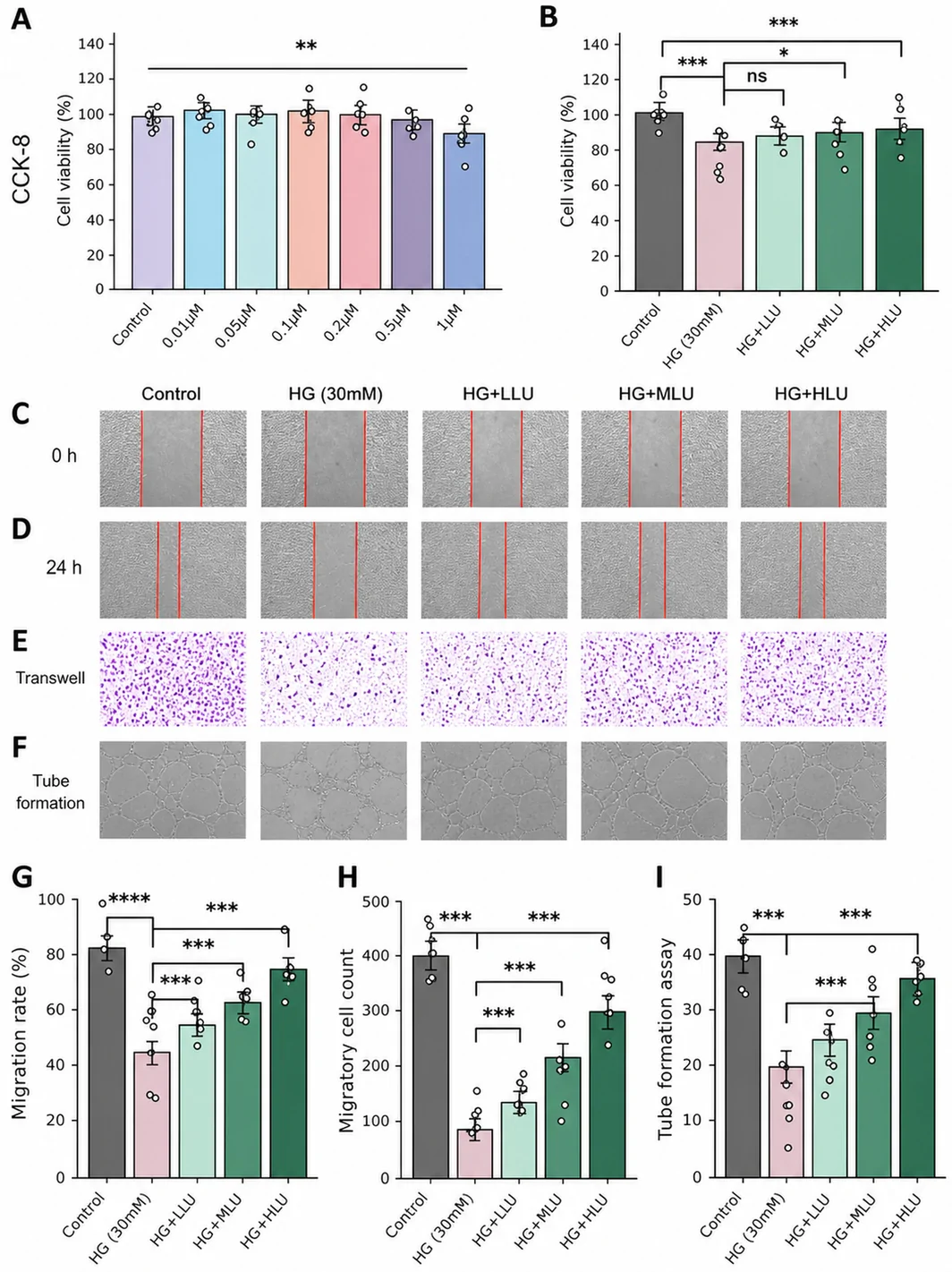
LU improves HUVEC viability, migration, and angiogenic capacity under high‐glucose conditions. (A) Screening of LU concentrations (0.01–1 μM) using the CCK‐8 cell‐viability assay. (B) HUVEC viability under normal‐glucose control conditions, high‐glucose exposure (HG; 30 mM), and high glucose combined with low‐, medium‐, or high‐concentration LU treatment. (C, D) Representative images of the scratch‐wound migration assay at 0 and 24 h. (E) Representative images of the Transwell migration assay. (F) Representative images of the Matrigel tube‐formation assay. (G) Quantification of scratch‐wound migration. (H) Quantification of migrated cells in the Transwell assay. (I) Quantification of tube formation. LU concentration‐dependently restored HUVEC viability, migration, and tube‐forming capacity impaired by high‐glucose exposure. Data are presented as mean ± SD from 3 independent experiments. Statistical comparisons were performed using one‐way ANOVA followed by Tukey's multiple‐comparison test. *p* < 0.05, *p* < 0.01, *p* < 0.001, and *p* < 0.0001; ns, not significant.

### 3.8 LU Increases Cell Migration and Encourages Angiogenesis in High‐Glucose Environments

Both have healing scratches. Transwell migration tests (Figure 6C,D,G) showed that the high‐glucose (HG, 30 mM) group significantly reduced HUVEC migration compared with the normoglycemic control group (*p* < 0.001). All tested LU concentrations markedly increased migration capacity in a dose‐dependent manner (*p* < 0.001 vs. the HG group).

Similarly, tube formation assays (Figure 6F,I) showed that high glucose conditions markedly suppressed the formation of capillary‐like structures (*p* < 0.001 vs. control). LU treatment significantly improved angiogenic potential, as evidenced by increased tube formation, also in a dose‐responsive manner. These findings indicate that LU counteracts high glucose‐induced endothelial dysfunction by promoting both migration and angiogenesis.

### 3.9 LU Enhances Endothelial Cell Viability via Activation of the Nrf2 Pathway

CCK‐8 assays were conducted using 0.2 μM LU, identified as the optimal concentration from earlier screenings. High glucose exposure significantly reduced cell viability compared to controls, *p* < 0.001(Figure 7A). LU treatment significantly improved cell survival (*p* < 0.001 vs. HG group). However, co‐treatment with the Nrf2 inhibitor ML385 markedly attenuated the pro‐survival effect of LU (*p* < 0.001 vs. LU group), suggesting that the beneficial effect of LU is at least partially mediated by the Nrf2 signaling pathway. These findings indicate that LU‐mediated cytoprotection is Nrf2‐dependent, while involvement of ferroptosis is inferred from downstream antioxidant and lipid peroxidation markers.

**FIGURE 7:**
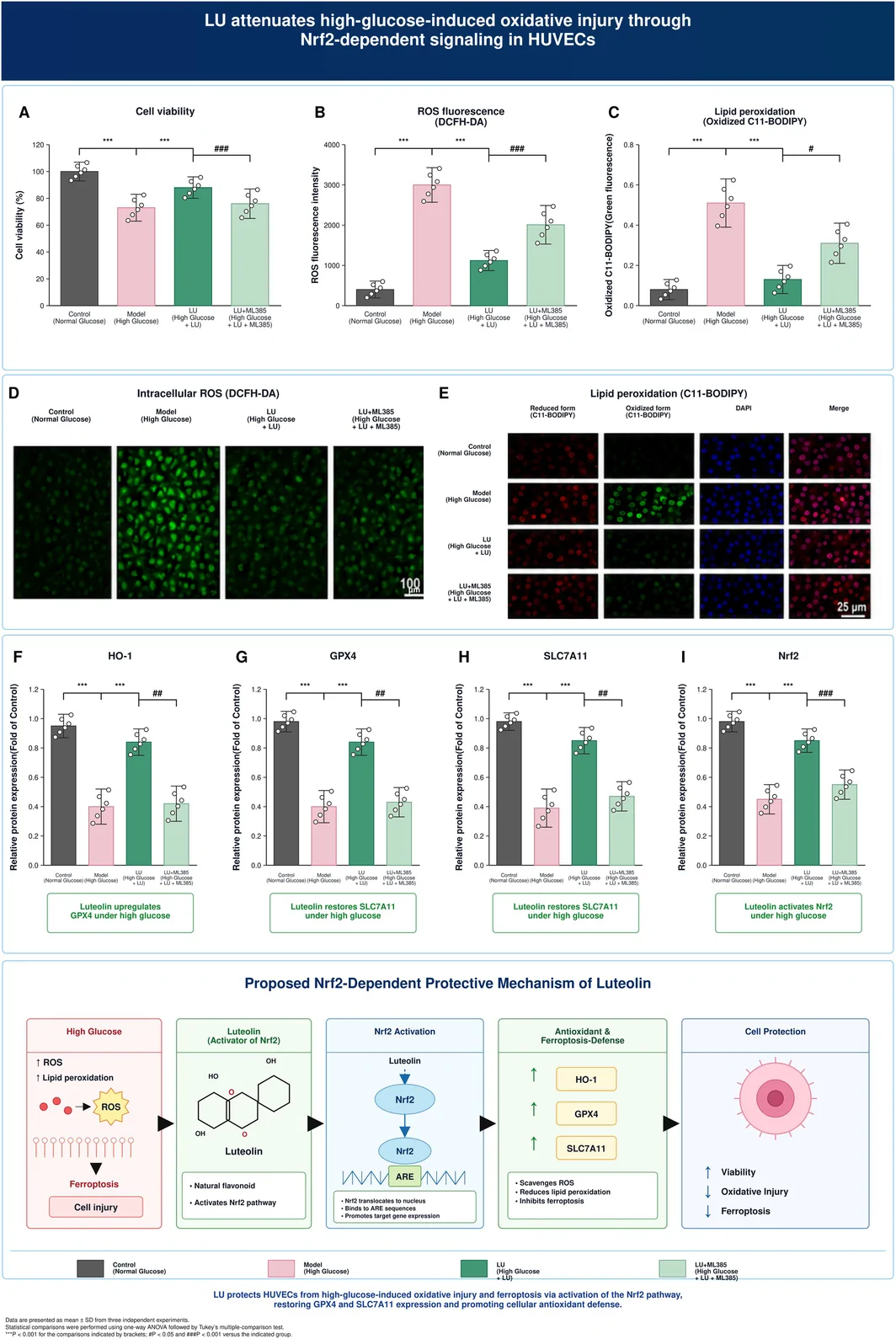
LU attenuates high‐glucose‐induced oxidative injury through Nrf2‐dependent antioxidant and ferroptosis‐defense signaling in HUVECs. (A) HUVEC viability under normal‐glucose control conditions, high‐glucose conditions, high glucose plus luteolin (LU), and high glucose plus LU in the presence of the Nrf2 inhibitor ML385, determined using the CCK‐8 assay. (B) Quantification of intracellular reactive oxygen species (ROS) fluorescence intensity measured using DCFH‐DA. (C) Quantification of lipid peroxidation based on oxidized C11‐BODIPY green fluorescence. (D) Representative fluorescence images of intracellular ROS detected by DCFH‐DA staining. (E) Representative fluorescence images of C11‐BODIPY staining showing the reduced probe (red), oxidized probe (green), DAPI‐stained nuclei (blue), and merged images. (F–I) Quantitative analysis of the relative expression of HO‐1, GPX4, SLC7A11, and Nrf2, respectively, normalized to the control group. LU significantly improved cell viability, reduced intracellular ROS accumulation and lipid peroxidation, and restored the relative expression of antioxidant‐ and ferroptosis‐related proteins under high‐glucose conditions. These protective effects were partially attenuated by co‐treatment with ML385, supporting the involvement of Nrf2 signaling. The lower schematic summarizes the proposed Nrf2‐dependent antioxidant and ferroptosis‐defense mechanism of LU in high‐glucose‐injured HUVECs. Data are presented as mean ± SD from three independent experiments (*n* = 3). Statistical analysis was performed using one‐way ANOVA followed by Tukey's multiple‐comparison test. ***p < 0.001 for the comparisons indicated by brackets; *p* < 0.05, ^##^ *p* < 0.01, and ^###^ *p* < 0.001 versus the LU‐treated group. Scale bars = 100 μm in panel D and 25 μm in panel E.

### 3.10 LU Reduces the Production of ROS Caused by High Glucose by Activating Nrf2

Examining immunofluorescence, intracellular ROS levels in the HG group were significantly higher than those in the controls (*p* < 0.001), as demonstrated in Figure 7A. Treatment with LU significantly reduced ROS accumulation (*p* < 0.001 vs. HG), while the addition of ML385 reversed this effect, resulting in elevated ROS levels. These results support the role of LU in reducing oxidative stress in endothelial cells via Nrf2 activation.

### 3.11 In High Glucose‐Damaged HUVECs, LU Lowers Lipid Peroxidation Through the Nrf2 Pathway

Assessment of lipid peroxidation using C11‐BODIPY fluorescence (Figure 7B,D) revealed intense green fluorescence in the HG group, indicative of elevated lipid peroxidation. Treatment with LU significantly decreased this signal, reflecting reduced lipid peroxidation. However, the addition of ML385 reversed the effect, leading to increased peroxidation levels. These findings suggest that LU mitigates lipid oxidative damage induced by hyperglycemia, and that this protective effect depends on Nrf2 signaling.

Network pharmacology identified Nrf2‐related signaling as a potentially important mechanism underlying the protective effects of luteolin (LU) in diabetic foot ulcer repair. Pathway enrichment analyses highlighted antioxidant‐ and ferroptosis‐related pathways, while molecular docking suggested potential interactions between LU and several key network‐derived targets. Consistent with these computational predictions, LU significantly improved HUVEC viability and reduced high‐glucose‐induced intracellular ROS accumulation and lipid peroxidation. Furthermore, the protective effects of LU were partially attenuated by the Nrf2 inhibitor ML385, supporting the involvement of Nrf2‐dependent antioxidant signaling in mediating the cytoprotective effects of LU.

### 3.12 LU Attenuates High‐Glucose‐Induced Oxidative and Lipid‐Peroxidation Injury Through an Nrf2‐Dependent Mechanism

High‐glucose exposure significantly reduced HUVEC viability and increased intracellular ROS accumulation and lipid peroxidation compared with the normoglycemic control group. LU treatment markedly improved cell viability and reduced both ROS fluorescence and oxidized C11‐BODIPY signals under high‐glucose conditions. Co‐treatment with the Nrf2 inhibitor ML385 partially reversed these protective effects, resulting in reduced cell viability and increased oxidative and lipid‐peroxidation injury relative to LU treatment alone.

These findings support the involvement of Nrf2 signaling in the cytoprotective effects of LU. Nevertheless, the results should be interpreted as supporting an Nrf2‐dependent protective mechanism against oxidative stress and ferroptosis‐related injury rather than providing definitive evidence of direct activation of the Nrf2–GPX4–SLC7A11 signaling pathway.

## 4 Discussion

The present study demonstrates that LU significantly accelerates repair processes in DFU models. The improvements observed included enhanced wound closure, preservation of tissue structure, increased collagen deposition, promotion of angiogenesis, and reduction of inflammatory and oxidative damage. These findings support the view that LU exerts multitarget protective effects during impaired wound healing. Mechanistic exploration indicated that LU achieves these benefits primarily through modulation of the Nrf2 pathway, which is closely linked to ferroptosis regulation. Inhibition of Nrf2 using ML385 largely abolished the protective responses, confirming the pathway's critical role in mediating LU's therapeutic activity.

The present study employed LU perchlorate rather than parent LU. This choice was based on formulation considerations, as the perchlorate salt offers improved stability and handling properties for topical application, without altering the core flavonoid structure responsible for biological activity. Thus, the observed therapeutic effects are attributable to LU itself, while the perchlorate form enhances experimental consistency and translational relevance for topical DFU treatment.

Prompt and effective closure of chronic ulcers is essential to reduce infection risk and lower the incidence of lower‐limb amputation (Lavery et al. 2024; Armstrong et al. 2017). In the present study, a well‐established diabetic foot ulcer model combining streptozotocin administration, dietary manipulation, and full‐thickness surgical wounding was employed, with recombinant human epidermal growth factor (rhEGF) serving as a reference treatment (Nie et al. 2020). Compared with untreated diabetic controls, LU‐treated animals exhibited significantly accelerated wound closure and improved healing kinetics. Notably, at later stages of repair, LU treatment resulted in more advanced tissue remodeling than rhEGF, as evidenced by enhanced re‐epithelialization and reduced inflammatory features. Histological analyses further corroborated these findings, revealing preserved epidermal architecture, more organized dermal collagen deposition, and attenuated inflammatory infiltration in LU‐treated wounds, consistent with previous reports describing the beneficial effects of natural compounds in diabetic wound healing (Xu et al. 2024).

Angiogenesis plays an indispensable role in the wound healing process. CD34 immunostaining is a widely used indicator of neovascularization and microvessel density (Díaz‐Flores et al. 2014; Fina et al. 1990). Our analysis revealed markedly higher CD34 expressions in LU groups compared to the model group, indicating a potent angiogenic effect. In parallel, LU modulated inflammatory and growth‐related cytokines. Specifically, ELISA assays demonstrated reduced TNF‐α and IL‐6 alongside increased VEGF, EGF, and PDGF, which are critical drivers of endothelial proliferation, keratinocyte migration, and tissue remodeling (Eming et al. 2014; Bao et al. 2009; Werner and Grose 2003; Barrientos et al. 2008; Gurtner et al. 2008). These changes confirm that LU not only suppresses inflammation but also facilitates regenerative processes required for effective wound repair.

The in vitro component of this study focused on human umbilical vein endothelial cells (HUVECs), which were selected to mechanistically evaluate angiogenesis‐related processes that are central to DFU repair. Nevertheless, diabetic wound healing is a complex, multicellular process involving keratinocytes, fibroblasts, immune cells, and macrophages in addition to endothelial cells. Therefore, the use of a single endothelial cell model represents a limitation of the present study. Future investigations incorporating additional skin‐resident cell types, co‐culture systems, or three‐dimensional wound models will be important to further elucidate the cell‐type–specific and intercellular effects of LU in the diabetic wound microenvironment.

The contribution of oxidative stress to the pathogenesis of diabetic foot ulcers is well established (Schäfer and Werner 2008; Eming et al. 2014). Chronic hyperglycemia leads to excessive accumulation of ROS, which promotes ferroptosis, a regulated form of cell death driven by iron‐dependent lipid peroxidation and failure of antioxidant defense systems (Stockwell et al. 2017; Wang et al. 2024). In diabetic wounds, depletion of cystine and glutathione compromises the activity of glutathione peroxidase 4 (GPX4), resulting in unchecked membrane lipid oxidation and subsequent cellular injury (Yang et al. 2014). This ferroptotic process impairs the viability of fibroblasts, keratinocytes, and endothelial cells, thereby disrupting granulation tissue formation and effective wound regeneration. Consistent with this mechanism, reduced GPX4 expression and activation of ferroptosis‐related pathways have been reported in diabetic wound tissues and experimental diabetic ulcer models, highlighting the pathological relevance of ferroptosis in non‐healing wounds (Zhang et al. 2025; Feng et al. 2022). In line with these observations, our study demonstrated marked ROS accumulation and lipid peroxidation in diabetic wounds, whereas LU treatment significantly attenuated oxidative damage both in vivo and in vitro.

In endothelial cell assays, LU significantly improved cell viability under high‐glucose conditions, enhanced migratory capacity, and promoted tube formation, indicating restoration of angiogenic function compromised by diabetic stress (Chen et al. 2020). In parallel, LU attenuated lipid peroxidation and reinforced cellular antioxidant defenses. At the molecular level, LU treatment upregulated the expression of glutathione peroxidase 4 (GPX4) and solute carrier family 7 member 11 (SLC7A11), two key regulators that limit ferroptosis by maintaining redox homeostasis. Consistent with this effect, LU activated nuclear factor erythroid 2–related factor 2 (Nrf2), leading to increased expression of downstream antioxidant targets, including heme oxygenase‐1 (HO‐1), GPX4, and SLC7A11 (Xiang et al. 2025). Importantly, pharmacological inhibition of Nrf2 with ML385 markedly attenuated the cytoprotective, pro‐angiogenic, and anti‐ferroptotic effects of LU, confirming that these actions are largely dependent on Nrf2 signaling.

Although the present study provides multiple lines of evidence supporting ferroptosis inhibition—including reduced lipid peroxidation, restoration of GPX4 and SLC7A11 expression, and attenuation of oxidative stress—it is important to acknowledge a limitation. The pharmacological inhibitor ML385 was employed to confirm the dependence of LU's protective effects on Nrf2 signaling; however, ML385 does not constitute a ferroptosis‐specific rescue agent. Therefore, while our data strongly suggest that LU suppresses ferroptotic injury through Nrf2‐mediated antioxidant defense, definitive confirmation using canonical ferroptosis inhibitors such as Ferrostatin‐1 or Liproxstatin‐1 was beyond the scope of the present study. Future investigations incorporating such agents will be necessary to further validate the ferroptosis‐specific contribution. In addition to endothelial‐driven angiogenesis, effective wound repair requires coordinated re‐epithelialization mediated by keratinocyte proliferation and migration. In this context, we previously demonstrated that the natural resin‐derived product sweetgum oil significantly enhances keratinocyte migration and proliferation in HaCaT cells, supporting the concept that plant‐derived bioactive compounds can promote wound healing through complementary, cell‐type–specific mechanisms (Yusuf et al. 2026).

Earlier and recent reports have shown that LU exerts beneficial effects on wound healing and tissue repair in diabetic models, largely attributed to general antioxidant and anti‐inflammatory properties (Li et al. 2025; Yang et al. 2024). Notably, the involvement of ferroptosis and its regulation by Nrf2 in diabetic foot ulcer healing was not examined in these studies.

Collectively, these findings delineate a multifaceted mechanism by which LU accelerates diabetic foot ulcer repair, encompassing suppression of oxidative stress, inhibition of ferroptosis, promotion of angiogenesis, and enhancement of extracellular matrix deposition (Figure 8). Through coordinated modulation of these interconnected processes, LU emerges as a promising candidate for DFU therapy. Notably, unlike previous studies that primarily focused on individual biological effects of LU, the present work provides integrative mechanistic evidence by combining network pharmacology, molecular docking, in vivo animal models, and targeted cellular assays with pharmacological inhibition. This comprehensive strategy strengthens the mechanistic framework underlying LU activity and highlights its translational potential.

**FIGURE 8:**
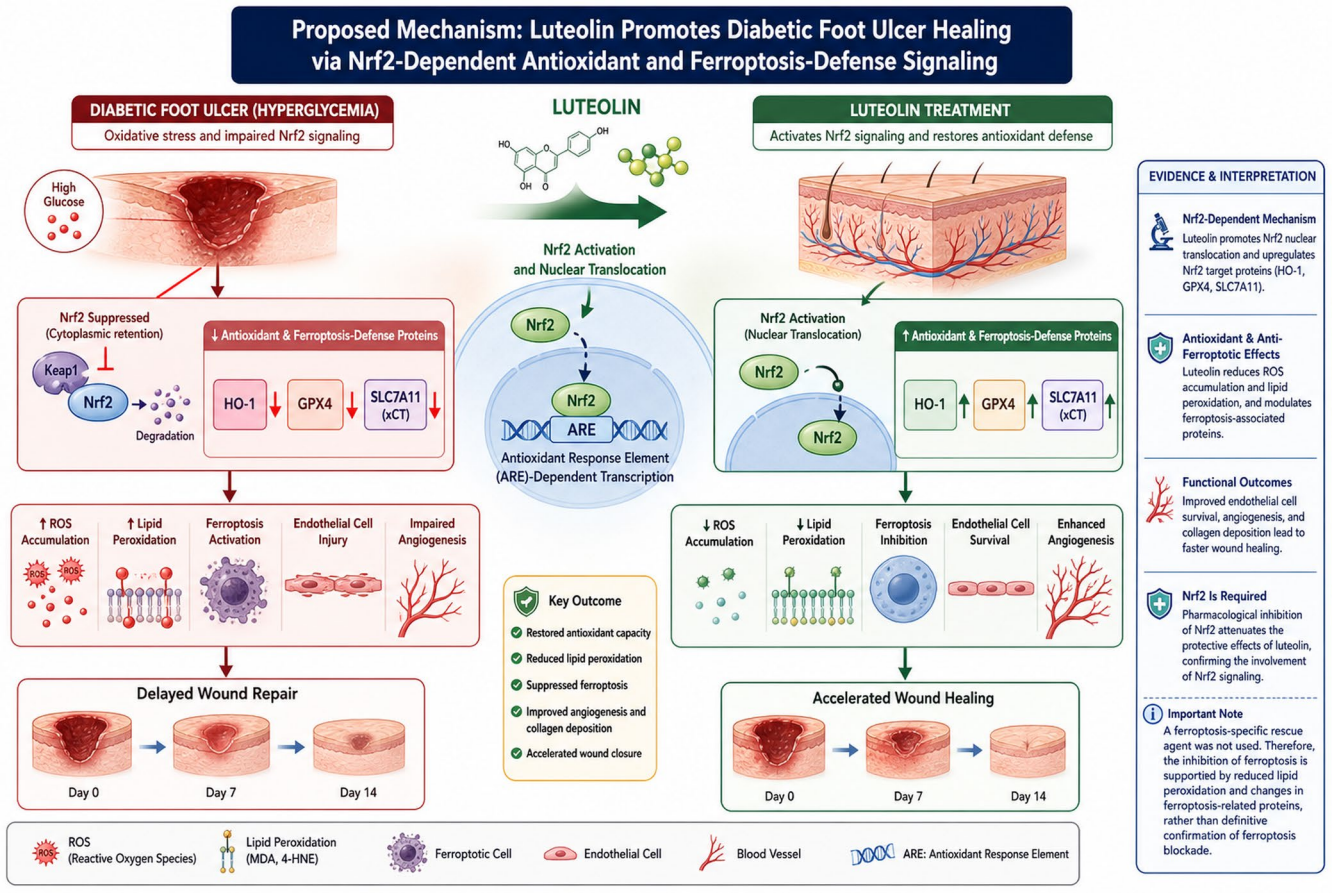
Proposed mechanism by which LU promotes diabetic foot ulcer healing through Nrf2‐dependent antioxidant and ferroptosis‐defense signaling. Hyperglycemia‐induced oxidative stress suppresses Nrf2 signaling in DFU wounds, resulting in reduced expression of HO‐1, GPX4, and SLC7A11; increased ROS accumulation and lipid peroxidation; ferroptosis‐related cellular injury; impaired angiogenesis; and delayed wound repair. LU activates Nrf2 signaling and promotes Nrf2 nuclear translocation and antioxidant response element‐dependent transcription, thereby restoring HO‐1, GPX4, and SLC7A11 expression. These effects reduce oxidative stress and lipid peroxidation, improve endothelial‐cell survival and angiogenesis, enhance collagen deposition, and accelerate wound closure. Pharmacological inhibition of Nrf2 with ML385 attenuated the protective effects of LU, supporting the involvement of Nrf2 signaling in the observed responses. Because a ferroptosis‐specific rescue agent was not used, the proposed inhibition of ferroptosis should be interpreted as a mechanism supported by lipid‐peroxidation measurements and ferroptosis‐associated protein expression rather than definitive confirmation of ferroptosis blockade.

## 5 Conclusion

In conclusion, the present study provides strong evidence that LU enhances diabetic foot ulcer repair through a multifaceted mechanism involving activation of Nrf2 signaling and suppression of ferroptosis. By integrating experimental validation in endothelial and animal models with network pharmacology and molecular docking analyses, we demonstrate that LU accelerates wound closure, promotes angiogenesis, improves extracellular matrix remodeling, and alleviates oxidative stress and inflammatory damage. Importantly, the attenuation of LU's protective effects by the Nrf2 inhibitor ML385 underscores the essential role of Nrf2 in mediating these responses.

Unlike previous studies that primarily emphasized the antioxidant or anti‐inflammatory properties of LU, this work provides comprehensive mechanistic evidence that LU functions as a regulator of ferroptosis in the context of diabetic wound healing. The dose‐dependent therapeutic responses observed in vivo, together with endothelial functional assays and pathway‐level analyses, highlight the translational potential of LU as a candidate agent for DFU management. Future studies should focus on pharmacokinetic characterization, long‐term safety evaluation, and potential synergy with clinically approved wound‐healing therapies to facilitate the advancement of LU from preclinical investigations toward clinical application.

## Author Contributions

**Ramiz Yusuf:** writing – review and editing, software, visualization. **Ayfer Busra Benkli:** investigation, writing – review and editing. **Muhammed Yusuf:** conceptualization, investigation, writing – original draft, methodology, validation, visualization, writing – review and editing, software, formal analysis, data curation, supervision, resources.

## Funding

The authors have nothing to report.

## Ethics Statement

All animal procedures were carried out in accordance with institutional guidelines for the care and use of laboratory animals and were approved by the relevant Animal Ethics Committee.

## Conflicts of Interest

The authors declare no conflicts of interest.

## Data Availability

The datasets generated and/or analyzed during this study are available from the corresponding author upon reasonable request.
